# The use of transvaginal synthetic mesh for anterior vaginal wall prolapse repair: a randomized controlled trial

**DOI:** 10.1007/s00192-013-2092-0

**Published:** 2013-04-30

**Authors:** Carlos A. Delroy, Rodrigo de A. Castro, Márcia M. Dias, Paulo C. Feldner, Maria Augusta T. Bortolini, Manoel J. B. C. Girão, Marair G. F. Sartori

**Affiliations:** Sector of Urogynecology and Vaginal Surgery, Department of Gynecology, Federal University of São Paulo, Rua Joaquim Floriano, 871 cj 92, São Paulo, SP Brazil 04534-000

**Keywords:** Colporrhaphy, Mesh, Pelvic organ prolapse, POP surgery, Anterior vaginal wall, Nazca TC™

## Abstract

**Introduction and hypothesis:**

The aim of the study was to compare the efficacy and safety of transvaginal trocar-guided polypropylene mesh insertion with traditional colporrhaphy for treatment of anterior vaginal wall prolapse.

**Methods:**

This is a randomized controlled trial in which women with advanced anterior vaginal wall prolapse, at least stage II with Ba ≥ +1 cm according to the Pelvic Organ Prolapse Quantification (POP-Q) classification, were randomly assigned to have either anterior colporrhaphy (*n* = 39) or repair using trocar-guided transvaginal mesh (*n* = 40). The primary outcome was objective cure rate of the anterior compartment (point Ba) assessed at the 12-month follow-up visit, with stages 0 and I defined as anatomical success. Secondary outcomes included quantification of other vaginal compartments (POP-Q points), comparison of quality of life by the prolapse quality of life (P-QOL) questionnaire, and complication rate between the groups after 1 year. Study power was fixed as 80 % with 5 % cutoff point (*p* < 0.05) for statistical significance.

**Results:**

The groups were similar regarding demographic and clinical preoperative parameters. Anatomical success rates for colporrhaphy and repair with mesh placement groups were 56.4 vs 82.5 % (95 % confidence interval 0.068–0.54), respectively, and the difference between the groups was statistically significant (*p* = 0.018). Similar total complication rates were observed in both groups, with tape exposure observed in 5 % of the patients. There was a significant improvement in all P-QOL domains as a result of both procedures (*p* < 0.001), but they were not distinct between groups (*p* > 0.05).

**Conclusions:**

Trocar-guided transvaginal synthetic mesh for advanced anterior POP repair is associated with a higher anatomical success rate for the anterior compartment compared with traditional colporrhaphy. Quality of life equally improved after both techniques. However, the trial failed to detect differences in P-QOL scores and complication rates between the groups.

## Introduction

It is believed that the incidence of pelvic organ prolapse (POP) will substantially increase in the next decades with the fast growth of the elderly population in developed countries [1]. It is estimated that 41 % of women aged 50–79 will present with some degree of POP at, with 35 % related to the anterior vaginal wall [2]. Recently, efforts have been made to establish the most appropriate surgical procedure to repair POP. The investigations are fundamentally based on the usage of synthetic prostheses in order to reestablish the pelvic floor anatomy and function as an alternative to standard native tissue repair [3].

The anterior vaginal wall is the most common compartment to prolapse and the major focus of the discussion about the potential benefits of augmentation with synthetic meshes [2, 4]. The last systematic review of the recent literature concluded that the use of meshes to repair the anterior compartment is associated with a better anatomical success rate when compared to anterior colporrhaphy (AC), although the authors highlighted the need for additional high-quality studies including both patient-reported and clinician-observed outcomes to support the use of mesh and to be able to verify its efficacy in the long term [5].

Even though rarely evidence-based [5], trocar-guided mesh kits have been increasingly used in POP surgery and involve the use of metal trocars for placement of a synthetic mesh, standardized in shape and size to support the vaginal walls. Nazca TC™ (Promedon, Córdoba, Argentina) is an example of a device designed to repair anterior vaginal wall prolapse. Palma et al. have published initial results of its usage. The authors prospectively analyzed a cohort of 104 patients and observed improved objective and subjective parameters after 1 year of follow-up [6]. The next logical step in research should be to confront this new technique with the current standard procedure.

In view of that, we developed this randomized controlled trial (RCT) intended to evaluate the efficacy and safety of both AC and the use of transvaginal synthetic mesh (Nazca TC™) to repair advanced anterior vaginal wall prolapse. We hypothesized that anterior repair by using the Nazca TC™ kit is not inferior to traditional colporrhaphy in 1-year follow-up time. We aimed to analyze and compare the objective success rate in the anterior compartment using point Ba measurements. Secondary endpoints were subjective improvement (condition-specific quality of life questionnaire), complication rate, and descent of all vaginal compartments [Pelvic Organ Prolapse Quantification (POP-Q)] after both surgical techniques.

## Patients and methods

The study was designed as a non-inferiority RCT comparing efficacy and complication rates of AC and trocar-guided transvaginal polypropylene mesh insertion (MESH) to repair advanced anterior vaginal wall prolapse. This study was conducted at the Sector of Urogynecology and Vaginal Surgery, Federal University of São Paulo, Brazil, a tertiary referral academic center, after the approval of the local Ethics Committee and registration at ClinicalTrials.gov (FDA) under protocol NCT00676325. The study was funded by the Federal University of São Paulo and Hospital São Paulo. Promedon contributed by donating the kits to be evaluated under the research protocol performed by urogynecology staff and fellows during regular activities, as part of their training program. No unrestricted research grant was provided.

From January 2007 to January 2009, women were assessed for eligibility by all authors during regular activities in the clinic. From among them, consecutive women presenting with anterior POP at least stage II beyond the hymen with point Ba equal to or greater than +1 according to the POP-Q classification [7] were initially enrolled as candidates for the study. We included patients presenting with either primary or recurrent POP cases, with the anterior compartment being the most prominent. We excluded women with malignant urogenital disease or previous pelvic radiotherapy, acute genitourinary infection, connective tissue disorders, systemic glucocorticoid treatment, insulin-treated diabetes, or clinical contraindications to a surgical procedure.

After being instructed and providing written informed consent to the first author, women who agreed to participate in the study were assigned to have either AC or MESH. The first author conducted the enrollment and randomization. Block randomization assigned in the ratio of 1:1 was done using a computerized random number generator using the program SPSS® (V17.0) at the moment of inclusion. A secretary blinded to the patients’ history and with no contact with the patients created envelopes that contained the allocation according to the order randomized by the computer. Once women were included in the trial, the intraoperative and postoperative protocol forms as well as the envelopes with the allocation group were attached in the patients’ files. So the surgeon was only aware of the allocation group in the operating room.

Sample size was calculated by the Diman® computer program on the basis of rates of anterior compartment objective measurements (point Ba) described in the literature: recurrence rate after AC (POP-Q ≥ stage II) ranging between 20 and 70 % and after synthetic mesh surgery in the anterior compartment ranging between 3 and 13 % [8–14]. Anatomical success rate for the anterior compartment was considered as 68 % for AC according to our center’s experience (data not published) and as 85 % for mesh repair, in order to evaluate the best scenario for AC and the worst scenario for mesh repair outcomes (17 % difference between them). We estimated that 35 subjects per group provides 80 % power to our model, with 0.05 significance level, and anticipating a 10 % loss to follow-up and/or dropout rate over the period of the study [15].

Statistical analyses were performed using the program SPSS® (V19.0). Descriptive analysis was presented in absolute numbers, mean and standard deviation for quantitative variables, while percentage was used for qualitative variables. Student’s *t* and Mann–Whitney tests were used to compare continuous variables between the groups. Chi-square and Fisher’s tests were used for evaluation of nominal variables. Analysis of variance (ANOVA) was performed for comparison of POP measurement between the study groups at pre- and postoperative time points. Per protocol, intention to treat, and number needed to treat analyses were planned. We considered a cutoff point of *p* < 0.05 for statistical significance in all analyses.

The study aimed to determine and compare the efficacy and safety of both surgical techniques after 1 year. The primary outcome was the objective evaluation of anatomical success, defined as anterior vaginal wall at stages 0 and I (Ba < −1) according to the POP-Q system. Secondary outcomes were the comparison of intra- and postoperative complications and the quality of life related to POP using the validated condition-specific prolapse quality of life (P-QOL) questionnaire. Total scores for each P-QOL domain range from 0 to 100 and are used to measure the severity of the POP symptoms [16]. In addition, we evaluated the other anatomical compartments using POP-Q measurements.

The pre- and postoperative protocol included: interview, urogynecological history, gynecological and general physical examination, urodynamics study when needed, blood test and urinalysis, and quality of life assessment using the P-QOL questionnaire validated for the Portuguese language [17]. POP symptoms such as vaginal “bulge,” pelvic pain, and sensation of heaviness were individually assessed using specific questions contained in the P-QOL. We considered as POP-positive symptoms if the patients answered “yes” (“slightly/a little,” “moderately,” or “a lot”) to at least one of the questions 3e-h. Dyspareunia was assessed by the unique question: “Do you have pain during intercourse?” and considered positive if the patient answered “yes.”

The patients were examined in the lying position with a full bladder and asked to perform the Valsalva maneuver. The descensus of the vaginal compartments was measured at the maximum straining point using a centimeter scale ruler. Total vaginal length was measured at rest under POP reduction with a vaginal speculum. Afterward, a straining examination in the standing position confirmed the full extent of the POP.

The surgeries were performed from February 2007 to December 2009. The AC procedure started with vaginal infiltration with a lidocaine and 2 % epinephrine solution diluted 1:1 in a total of 40 ml. A longitudinal midline incision of the vaginal mucosa from 2 cm of the urethral meatus to the uterine cervix or vaginal vault was performed and dissected away from the pubocervical fascia laterally and bilaterally. Purse string sutures were used to plicate the fascia with Vicryl® 0, followed by vaginal mucosa trimming and midline closure with interrupted suture using Vicryl® 2–0.

For the transvaginal mesh insertion, we used the trocar-guided kit Nazca TC™ (Promedon, Córdoba, Argentina) designed for anterior POP repair. It consists of a type I monofilament and macroporous polypropylene mesh. The set also contains one prepubic and two transobturator needles with removable, ergonomic handles. The surgical technique was previously described [6]. Briefly, after vaginal infiltration with lidocaine and vasoconstrictor solution, two 5-mm suprapubic incisions were made 3 cm apart. A full thickness vaginal incision from the midurethra towards the uterine cervix or vault was made allowing proper vaginal dissection extended towards the ascending branch of the ischium and inferior aspect of the pubic bone. Next, the prepubic needle was introduced through the vaginal incision and directed towards the corresponding suprapubic incision, and then the mesh arms were delivered bilaterally. The transobturator needle was inserted at the genitofemoral fold on an outside-in direction exiting closest to the ischial spine in the vaginal opening. Next, the transobturator arm was connected to the needle and was brought through the bilateral genitofemoral skin incision in a reverse fashion. Sutures were placed on the body of the mesh to the remnants of the cardinal ligament or the pericervical ring using polypropylene sutures to avoid apical cystocele recurrence. All four arms were gently pulled in a tension-free manner and trimmed if needed. The vaginal wall was closed using the Montgomery overlapping technique to avoid superposition of the suture line on the mesh with interrupted sutures using Vicryl® 2–0.

All procedures were conducted under spinal anesthesia. Cystoscopy was performed in the operating room at the surgeon’s discretion. All patients received cefazolin (2 g) and metronidazole (500 mg) as antibiotic prophylaxis. The procedures were performed by experienced surgeons (CAD, MMD, and RAC). Patients had their 14 F Foley vesical catheter and vaginal tampon removed on the first postoperative day (PO #1). A blood test was performed 24 h after the procedure to be able to detect an eventual drop in hemoglobin. The discharge occurred according to individual clinical conditions.

A social assistance worker routinely contacted the participants monthly by phone call, reinforcing the adherence to the treatment. The follow-up appointments occurred at day 7 and 1, 3, 6, and 12 months after surgery. In order to decrease examiner interference in our results, postoperative evaluations were conducted by a urogynecology physician blinded to the surgical procedure. On those occasions, anamnesis and physical examination with POP quantification were performed. Urinalysis was ordered if the patients presented with irritative urinary symptoms to rule out urinary tract infection. We considered urinary tract infection related to surgery if it occurred up to 30 days after surgery. Urinary retention and voiding dysfunction were considered if the patient was unable to void properly (post-void residual > 150 ml) at PO #1. Increased bleeding was characterized by a rate of hemoglobin drop equal to or greater than 2.0 g/dl 24 h after the procedure. The impact of the surgery on quality of life was assessed by P-QOL at the time of the 1-year follow-up.

## Results

**Fig. 1:**
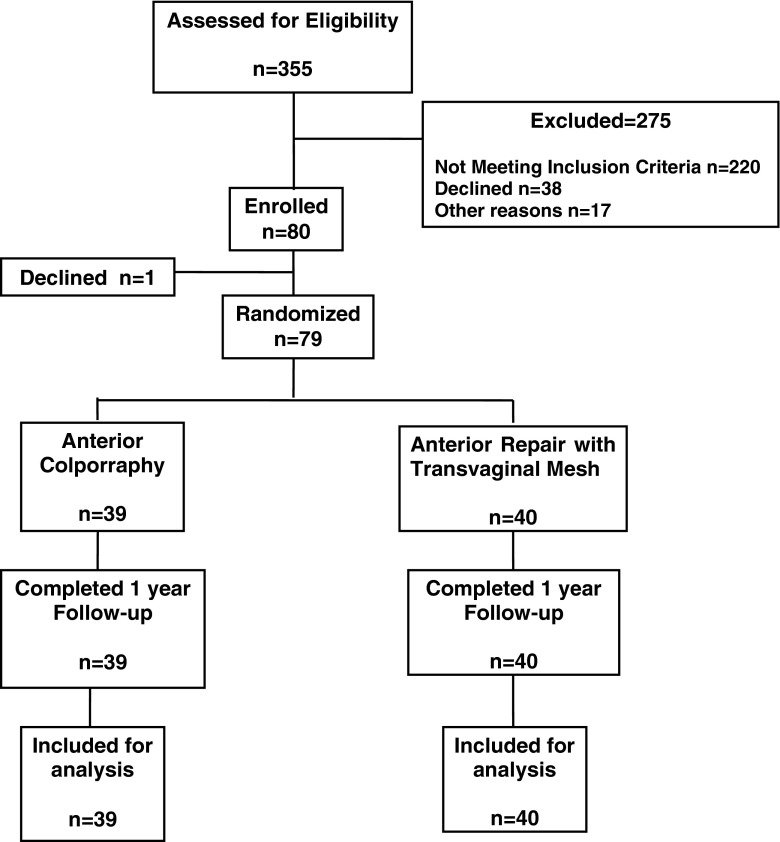
CONSORT diagram of participants. Note that data are available from the total cohort of randomized patients at the 1-year follow-up

During the study period, 355 consecutive women were assessed for eligibility. Eighty women met the inclusion criteria and were initially enrolled. From among them, 79 agreed to participate in the study with 39 (49.4 %) randomized to receive AC and 40 (50.6 %) MESH. All patients returned for follow-up at 1 year and had all protocol steps completed (Fig. 1).

Demographic and clinical pre- and postoperative parameters are shown in Table 1. The groups were homogeneous regarding age, with the majority in the postmenopausal phase, as well as body mass index (BMI), vaginal parity, and previous pelvic surgeries. In the AC and MESH groups, 30.8 and 20 % were recurrent POP cases, respectively. Both groups were also similar in relation to preoperative POP stage quantification for all different compartments. Of the patients, 20/39 (51.3 %) and 26/40 (65 %) presented with stage III anterior compartment prolapse in the AC and MESH groups, respectively.

**Table 1:** Pre- and postoperative demographic and clinical data of the study groups

Variable	AC (*n* = 39)	MESH (*n* = 40)	*p* value
Mean age, years (± SD)	59.6 (±10)	62.1 (±8.3)	0.231
Mean BMI, kg/m^2^ (± SD)	27.3 (±3.7)	27.6 (±4.7)	0.743
Mean vaginal parity (range)	4 (2–6)	5.3 (0.7–9.9)	0.314
Previous POP surgery, *n* (%)	13 (33.3 %)	8 (20 %)	0.180
Previous hysterectomy, *n* (%)	3 (7.6 %)	1 (2.5 %)	0.099
Previous SUI surgery, *n* (%)	12 (30.8 %)	8 (20 %)	0.271
Menopausal status, *n* (%)			
Premenopausal	7 (17.9 %)	2 (5.0 %)	0.087
Postmenopausal	32 (82.1 %)	38 (95 %)	
Anterior POP-Q stage, *n* (%)			
II	16 (41.0 %)	8 (20 %)	0.099
III	20 (51.3 %)	26 (65.0 %)	
IV	3 (7.7 %)	6 (15.0 %)	
Posterior POP-Q stage, *n* (%)			
0/I	9 (23 %)	18 (45 %)	0.083
II	28 (71.8 %)	20 (50 %)	
III	2 (5.1 %)	2 (5 %)	
Apical POP-Q stage, *n* (%)			
0/I	31 (79.5 %)	28 (70 %)	0.07
II	3 (7.7 %)	9 (22.5 %)	
III	5 (12.8 %)	3 (7.5 %)	
Concomitant surgical procedures, *n* (%)			
Vaginal hysterectomy/trachelectomy	14 (35.9 %)	8 (20 %)	0.115
Enterocele repair	3 (7.6 %)	4 (10 %)	0.91
Site-specific posterior colporrhaphy	28 (100 %)	17 (42.5 %)	0.83
Sacrospinal fixation	0	1 (2.5 %)	0.97
Mean operative time, min (± SD)	46 (±28.1)	99.1 (±35.8)	<0.001*
Mean length of hospitalization, days (± SD)	3.3 (±1.2)	3.2 (±2.6)	0.85
Postoperative adverse events, *n* (%)			
Intraoperative			
Increased bleeding	12 (30.8 %)	18 (45.0 %)	0.193
Blood transfusion	1 (5.1 %)	2 (5 %)	1.00
Bladder perforation	0	0	–
Urethral perforation	0	1 (2.5 %)	0.99
Postoperative	0	0	–
Tape exposure	0	2 (5 %)	0.76
Wound infection	0	0	–
Urinary retention	2 (5.1 %)	1 (2.5 %)	0.88
Voiding dysfunction	0	1 (2.5 %)	0.99
UTI	5 (13.8 %)	8 (20 %)	0.34
Dyspareunia	4 (10.2 %)	2 (5 %)	0.78
Transient thigh numbness	0	1 (2.5 %)	0.99

Student’s *t* test, Mann–Whitney test, Pearson’s chi-square test, and Fisher’s test. A significant difference is indicated by *p < 0.05

*SUI* stress urinary incontinence, *UTI* urinary tract infection

With regard to surgical procedures, the mean operative time was significantly longer in the MESH group (99.1 min) compared to the AC group (46 min) (*p* < 0.001). However, the length of hospitalization was not different between them. As expected, concomitant other pelvic reconstructive procedures were relatively common in both groups (*p* > 0.05).

**Fig. 2:**
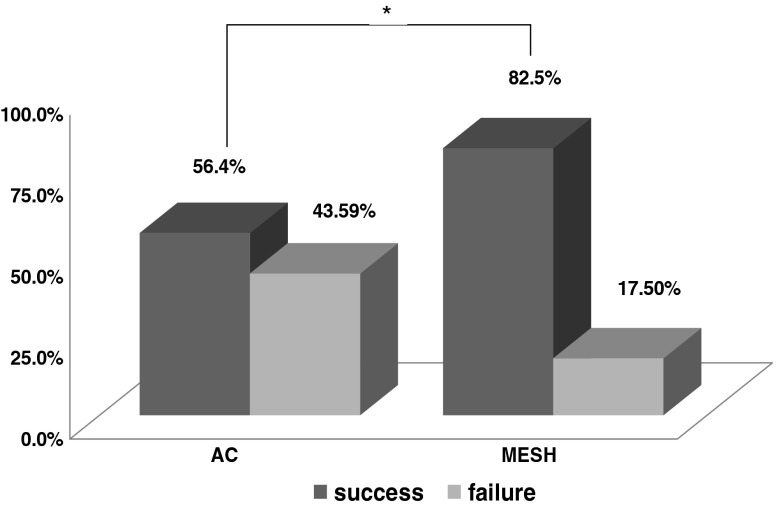
Anatomical success defined as point Ba < −1. Values are given in % of patients that met the cure criteria from each group. Pearson’s chi-square test. A significant difference is indicated by *p < 0.05

Per protocol and intention to treat analyses evidenced that around 82.5 % of the patients from the MESH group and 56.4 % from the AC group met the strict criteria for anatomical success in the anterior compartment (95 % confidence interval 0.068–0.54) at the 1-year follow-up, and this difference showed statistical significance (*p* = 0.018) (Fig. 2). The mean preoperative point Ba was +2.22 cm in the AC patients and +2.77 cm in the patients that received MESH; mean postoperative values were −1.44 and −1.97 cm, respectively (Table 2). The number needed to treat (NNT) was calculated as 4.

**Table 2:** Anatomical objective measurements at the 1-year follow-up

			AC			MESH			
Point	Moment	Interaction	*n*	Mean	SD	*n*	Mean	SD	*p* ^b^ value
	Pre-op		39	1.7	1.0	40	2.0	0.8	0.769
Aa	Post-op	*p* ^i^ = 0.114	39	−1.7	0.9	40	−1.9	1.0	
				*p* ^a^ < 0.001*			*p* ^a^ < 0.001*		
	Pre-op		39	2.3	1.5	40	2.8	1.3	0.072
Ba	Post-op	*p* ^i^ = 0.004*	39	−1.4	1.0	40	−1.9	1.1	0.018*
				*p* ^a^ < 0.001*			*p* ^a^ < 0.001*		
	Pre-op		39	−3.4	2.8	40	−3.2	2.0	0.615
C	Post-op	*p* ^i^ = 0.742	39	−4.8	1.2	40	−4.1	1.6	
				*p* ^a^ <0.001*			*p* ^a^ <0.001*		
	Pre-op		39	4.3	1.0	40	5.0	1.2	0.005*
gh	Post-op	*p* ^i^ = 0.909	39	2.8	0.9	40	3.3	1.0	
				*p* ^a^ < 0.001*			*p* ^a^ < 0.001*		
	Pre-op		39	2.7	0.7	40	3.0	0.9	0.119
pb	Post-op	*p* ^i^ = 0.013*	39	3.6	0.8	40	3.5	0.8	0.232
				*p* ^a^ < 0.001*			*p* ^a^ < 0.001*		
	Pre-op		39	7.1	0.8	40	6.8	1.0	0.31
tvl	Post-op	*p* ^i^ = 0.106	39	7.2	1.2	40	6.8	1.3	
				*p* ^a^ < 0.001*			*p* ^a^ < 0.001*		
	Pre-op		39	−0.6	1.5	40	−1.2	1.2	0.071
Ap	Post-op	*p* ^i^ = <0.001*	39	−2.4	0.6	40	−1.5	1.2	< 0.001*
				*p* ^a^ < 0.001*			*p* ^a^ = 0.318		
	Pre-op		39	−1.0	1.0	40	−1.4	0.9	0.059
Bp	Post-op	*p* ^i^ = <0.001*	39	−2.4	0.7	40	−1.5	1.2	< 0.001*
				*p* ^a^ <0.001*			*p* ^a^ = 0.636		
	Pre-op		38	−5.5	1.9	33	−4.7	1.3	0.001*
D	Post-op	*p* ^i^ = 0.556	24	−6.3	0.8	24	−5.6	1.3	
				*p* ^a^ < 0.001*			*p* ^a^ < 0.001*		

*gh* genital hiatus, *pb* perineal body, *tvl* total vaginal length

ANOVA test: *p* ^a^ values obtained from intragroup analyses according to the time (preoperative mean point versus postoperative mean point); *p* ^b^ values obtained from intergroup postoperative analyses (AC postoperative mean point versus MESH postoperative mean point); *p* ^i^ values obtained from the analyses of interaction between groups and moments (four mean points: preoperative AC versus postoperative AC versus preoperative MESH versus postoperative MESH). Significant difference is indicated by *p < 0.05. Note that all point measurements of both groups significantly improved in the postoperative time when compared to preoperative status. Note that a positive interaction factor was present in the analyses of points Ba, PB, Ap, and Bp. Note that final analyses showed significantly different values for the anatomical points Ba, gh, Ap, Bp, and D between the study groups

As shown in Table 2, we observed higher measures of POP-Q points in the postoperative compared to the preoperative time in both groups (*p* < 0.001 for all compartments). Some analyses showed an interaction effect between the measurements in the two time points (pre- and postoperative) and the two groups. In those situations, an ANOVA test was required and evidenced an additional statistically significant difference in the points gh, Ap, Bp, and D, favoring the AC group for the points Ap and Bp (*p* < 0.001 for both) and favoring the MESH group for gh and D (*p* = 0.005 and 0.001, respectively). Similar measurements were observed for Aa, C, and tvl between the groups.

By evaluating specific questions from the P-QOL questionnaire related to POP symptoms, we could find evidence that ten (26 %) patients from the AC group reported at least one bothersome POP symptom 1 year after surgery. Among them, eight have received further revision with mesh repair, and two opted for another AC. On the other hand, two (5 %) patients from the MESH group were symptomatic after 1 year but did not want another surgical procedure. Women presenting with recurrent POP symptoms had POP stage II (Ba = 0 or +1) during physical examination.

Intra- and postoperative total complication rates were 30.8 % (12/39) and 45 % (18/40) for the AC and MESH groups, respectively. Adverse events are described in Table 1. We had increased bleeding (hemoglobin drop < 2 g/dl) in two patients from the MESH group and one from the AC group, with no blood transfusion required. Bladder perforation due to digital dissection occurred in one surgery using mesh, detected and repaired intraoperatively, with no ureteral damage by cystoscopy. This woman had an indwelling catheter for 14 days, without developing any further voiding dysfunction. Postoperative complications were similar in both groups. Of 39 patients in the AC group, 19 were sexually active prior to surgery and also after the procedure. Among them, four reported dyspareunia at the 1-year follow-up (21 %). Regarding the MESH group, 21/40 women were sexually active before the operation, and 2 of them described pain during intercourse after the surgery (10 %). In addition, two patients resumed their sexual life after anterior repair with mesh augmentation. Mesh extrusion was noted in 2/40 (5 %). They were smaller than 1 cm and asymptomatic. After failure of conservative local estrogen therapy for 30 days, the portion of the extruded meshes were trimmed under local anesthesia in the clinic.

Subjective results by the analyses of the P-QOL questionnaire showed improvement in all nine domains of the questionnaire 1 year after repair with either AC or MESH (*p* < 0.001 for all). When compared between the techniques, the domain scores were not different between the groups, suggesting that the efficacy of both procedures was equal according to the patients’ reports (Table 3).

**Table 3:** Comparison between the study groups of pre- and 1-year postoperative P-QOL scores

P-QOL questionnaire domains	AC	MESH	*p* value
General health perceptions			
Pre-op	42.2 ± 21.2	46.79 ± 22.33	0.38
Post-op	24.1 ± 10.5	26.28 ± 21.30	0.98
Prolapse impact			
Pre-op	79.3 ± 30.0	74.35 ± 33.41	0.33
Post-op	3.4 ± 10.3	3.41 ± 15.06	0.70
Role limitation			
Pre-op	64.9 ± 25.7	45.72 ± 39.35	0.78
Post-op	2.8 ± 15.4	0.0 ± 0.0	0.33
Physical limitation			
Pre-op	63.7 ± 25.2	55.55 ± 8.64	0.80
Post-op	2.8 ± 15.4	2.13 ± 7.53	0.46
Social limitation			
Pre-op	34.8 ± 20.0	36.60 ± 13.52	0.09
Post-op	0.0 ± 0.0	0.56 ± 13.03	–
Personal relationship			
Pre-op	54.1 ± 34.4	27.77 ± 12.96	0.61
Post-op	0.0 ± 0.0	0.0 ± 0.0	0.35
Emotions			
Pre-op	69.3 ± 30.0	59.54 ± 12.36	0.06
Post-op	1.1 ± 6.1	1.13 ± 0.0	0.95
Sleep/energy			
Pre-op	48.2 ± 22.4	31.26 ± 11.82	0.68
Post-op	4.0 ± 11.4	10.68 ± 6.93	0.50
Severity measures			
Pre-op	42.8 ± 20.0	28.84 ± 11.35	0.19
Post-op	1.1 ± 4.8	1.06 ± 16.87	0.20
	*p* = 0.001*	*p* = 0.001*	

Values are given as absolute numbers. ANOVA test. A significant difference is indicated by *p < 0.05. Note that all questionnaire domains of both groups significantly improved in the postoperative time when compared to preoperative status. Note that final analyses showed no difference in P-QOL scores between the study groups

## Discussion

A recent systematic review article acknowledges that mesh insertion may have a role in reconstructive pelvic surgery in women [18]. The last meta-analysis of some high-quality RCTs supports the use of transvaginal synthetic meshes over native tissues for anterior vaginal wall prolapse repair considering anatomical results [5]. On the other hand, there are still relevant questions raised by the scientific community about its utilization due to potential risks inherent to the procedure as well as which patients would benefit from mesh augmentation [18–20]. However, there seems to be a consensus that additional comparative studies are required to clarify those issues.

Very few studies have described the results of trocar-guided commercial mesh kits for anterior compartment prolapse under the auspices of an RCT [11, 14, 20], which motivated us to perform this study evaluating Nazca TC™. Our results are in accordance with the majority of previous studies involving polypropylene meshes in general [9, 11, 13, 21] and have shown that the anatomical success rate is higher by using mesh, with no difference regarding quality of life related to POP and complication rates when AC was compared to mesh augmentation in advanced anterior POP at the 1-year follow-up. However, it is difficult to compare the findings of each study with other trials owing to variations in the surgical procedures, implant materials, outcomes measurements, and objective cure criteria [3, 20]. Also, the mesh anchoring mechanism varies among the studies, with the armed transobturator system used in some commercial kits. With those, one may advocate that they are different procedures and should not be equally compared.

We observed an absolute difference in the objective success rate of around 26 % between the techniques, favoring the mesh group. In a large multicenter trial involving 389 patients, Altman and coworkers evaluated another trocar-guided transvaginal synthetic mesh kit for anterior POP repair and reported a 26 % point difference, which strengthens our findings [21]. We estimate that the NNT is 4, meaning that one POP recurrence is avoided in each 4 patients that receive mesh augmentation for anterior compartment repair.

The literature analyses should also take into account other relevant aspects such as POP stages, concomitant surgical procedures, and recurrent cases enrolled in each trial [3]. Those may interfere with interpretation of the results. In this RCT, we strictly included advanced anterior POP cases and the groups were homogeneous regarding the most important demographic data including the ones related to previous and associated surgeries. However, our study involved primary as well as recurrent POP and we were not able to statistically analyze them separately.

We had a 5 % rate of mesh extrusion, which is in agreement with the range described in the literature (3–19 %) [5, 18, 21]. We postulate that the vaginal mucosa closure in an overlapping fashion may explain at least in part the low rate of extrusion, an idea that must be a subject for further studies.

We observed better posterior compartment anatomical findings (points Ap and Bp) as well as a higher rate of dyspareunia in the AC group. Those may represent the results of the additional posterior colporrhaphy repair that patients from this group have received, even though this study involved a limited number of participants making it difficult to derive precise conclusions considering concomitant procedures. Based on our results, it is interesting to note the lack of apical support provided by this trocar-guided mesh kit, not designed to treat associated advanced apical prolapse.

The International Continence Society considers POP-Q stages 0 and I as anatomical success [22]. However, this definition has been questioned when considering treatment outcomes, since around two thirds of multiparous women present with some degree of POP, and the majority are asymptomatic [23]. Moreover, patients may not be aware of prolapse stage II [24]. Therefore, cure criteria should be more properly assessed by adding the evaluation of symptoms reported by the patients [5, 24]. In our study, we used the validated condition-specific P-QOL questionnaire to evaluate the patient’s bothersome symptoms related to POP [17]. Both techniques used to repair anterior compartment prolapse significantly improved postoperative quality of life compared with preoperative status. No significant differences were detected in the scores of all questionnaire domains in the postoperative assessment between AC and repair using mesh.

Our trial achieved high rates of follow-up. Some aspects may have accounted for the treatment adherence: the small number of participants, positive reinforcement by phone calls throughout the trial, and the fact that our population is composed of humble women who are dependent on the public health system to receive good care.

In conclusion, our study demonstrated that transvaginal synthetic mesh (Nazca TC™) for advanced anterior POP repair is associated with a higher anatomical success rate of the anterior compartment compared with AC using native tissues. Quality of life equally improved after both techniques. However, the trial failed to detect differences in subjective cure rates, quality of life, or complication rates between the groups at the 1-year follow-up. With that in mind, additional studies involving a larger population and a longer follow-up time are required to determine the role of the trocar-guided synthetic mesh kits in pelvic floor reconstruction.
